# Supplementary material for: A scoping review of patient-centred tuberculosis care interventions: Gaps and opportunities
Source: PLOS Glob Public Health. 2023 Feb 2;3(2):e0001357. doi: 10.1371/journal.pgph.0001357 (PMC10021744; doi:10.1371/journal.pgph.0001357)
Supplement: S2 Appendix — (DOCX) [file pgph.0001357.s002.docx]

**S2 Appendix: full electronic search strategy for the PubMed database**

We searched on the PubMed database using multiple keyword combinations related to patient-centred care and tuberculosis as outlined in Table 1. We limited the search to between January 2005 and March 2020 (with the most recent search conducted on 23 March 2020) and to English-language manuscripts.

Table 1 – Full search strategy on PubMed database

| PubMed search terms* | Number of records |
| --- | --- |
| tuberculosis AND “patient-centred” | 85 |
| tuberculosis AND “patient-centered” | 160 |
| tuberculosis AND “patient-centric” | 9 |
| tuberculosis AND “patient-focused” | 6 |
| tuberculosis AND “patient-oriented” | 5 |
| tuberculosis AND “patient-orientated” | 2 |
| tuberculosis AND “people-centered” | 3 |
| tuberculosis AND “people-centred” | 6 |
| tuberculosis AND “person-centred” | 5 |
| tuberculosis AND “person-centered” | 3 |
| tuberculosis AND “family-centred” | 3 |
| tuberculosis AND “family-centered” | 2 |
| tuberculosis AND “social support” | 235 |
| tuberculosis AND “socio-economic support” | 7 |
| tuberculosis AND “economic support” | 22 |
| tuberculosis AND enablers AND care | 300 |

*Limits and filters applied:

- Date range: 01 January 2005 – 23 March 2020
- Language: English
