# Supplementary material for: A scoping review of patient-centred tuberculosis care interventions: Gaps and opportunities
Source: PLOS Glob Public Health. 2023 Feb 2;3(2):e0001357. doi: 10.1371/journal.pgph.0001357 (PMC10021744; doi:10.1371/journal.pgph.0001357)
Supplement: S3 Appendix — DR-TB = drug-resistant TB. SAT = self-administered therapy. XDR-TB = extensively drug-resistant TB. LTFU = lost to follow-up. DOT = directly observed therapy. (DOCX) [file pgph.0001357.s003.docx]

**S3 Appendix. Full text research articles included in the review, grouped by implementation of patient-centred TB care.**

|  | **First author, year** | **Setting/**  **Country** | **Design** | **Implementation of patient-centred TB care** |
| --- | --- | --- | --- | --- |
| **Emotional and psychosocial support** | Chalco et al. 2006 | Peru | Ethnographic study | Emotional support provided by community nurses to people with DR-TB, including counselling and gestures of support and warmth, routine home visits, and organised group meetings to support mental health. |
|  | Acha et al. 2007 | Peru | Qualitative methods | A psychosocial support group intervention for people with DR-TB, including support groups, recreational excursions, symbolic celebrations, and family workshops. The intervention aimed to improve adherence. |
|  | Khanal et al. 2017 | Nepal | Qualitative methods with intervention development | Development of a psychosocial support intervention for DR-TB informed by collaboration with people affected by TB, their family members, health workers, and the National TB Programme. Important recommendations for the intervention were the need for clear information provided by health workers, clearly defined referral routes to manage side effects, privacy during treatment, home administration of treatment, greater involvement of family members, and community campaigns to reduce stigma. |
|  | Walker et al. 2018 | Nepal | Mixed methods study | A psychosocial support package for people with DR-TB which included educational materials on DR-TB for patients and family members, screening for depression, and referral for counselling if needed. A support group was also available to patients. The study tested the feasibility and acceptability of the intervention and found high acceptability among patients, albeit with feasibility challenges for implementation in the National TB Programme. |
|  | Li et al. 2018 | China | Community-based repeat measurement trial | A comprehensive social support intervention for elderly people affected by TB that included the following components: health education, psychotherapy delivered at home, and family and community support by way of home-visits, psycho-educational workshops, and peer support. The primary outcome was the social support level experienced by people affected by TB. |
|  | Adepoyibi et al. 2019 | Papua New Guinea | Pilot intervention study | A patient education and counselling model of care for people with DR-TB focusing on emotional support, education, and referral to external social services to improve treatment outcomes. Counselling sessions were grouped into standard sessions for all patients timed to occur at critical stages of treatment, preventive therapy sessions, special sessions to address for example SAT, diagnosis of XDR-TB, treatment interruption, and initiation of new drugs, and adherence planning sessions. Visual aids and board games were used as educational tools for children and adolescents. |
| **Socio-economic relief** | Baral et al. 2014 | Nepal | Pilot intervention study | People with DR-TB received either counselling alone, or both counselling and financial support to assess the impact of these interventions on treatment success. |
|  | Bhatt et al. 2019 | India | Retrospective cohort study | An integrated psycho-socio-economic support package for DR-TB patients on second line anti-TB treatment, which included nutritional supplements, socio-economic support in the form of cash handouts, and psycho-emotional support, as needed. Impact was measured on treatment outcomes using routine data. |
|  | Fuady et al. 2019 | Indonesia | Mixed methods study with simulation of hypothetical scenarios | The study measured the socio-economic impact of TB and DR-TB and assessed patients’ needs for social protection. Using these data, they developed eight scenarios for financial support, and measured the impact of each scenario on the incidence of catastrophic costs. |
| **Enhanced support for patients at risk of LTFU** | Gelmanova et al. 2011 | Russia | Comparative study | A patient-centred TB treatment delivery programme (“Sputnik”) for patients at high risk of becoming LTFU. The programme included a high nurse-to-patient ratio, more staff time per patient to facilitate bonding and defaulter searching, and easier access to specialists and expanded social and psychological support. |
|  | Snyman et al. 2018 | South Africa | Qualitative study | A treatment interruption intervention aimed at people with DR-TB receiving treatment either through DOT or SAT, and who are at risk of becoming LTFU. The intervention included home visits to understand reasons for treatment interruption, screening of substance abuse and mental illness, and the design of individualised management plans by a multidisciplinary team. |
| **Decentralised TB care** | Brust et al. 2012 | South Africa | Pilot implementation | Implementation of a home-based treatment programme for people with DR-TB to test feasibility, safety, and impact on treatment outcomes. The programme was delivered by nurses and community health workers who provided daily injections and adherence support, monitored adverse events, and educated patients and family members on infection control practices. |
| **Stigma reduction interventions** | Macq et al. 2008 | Nicaragua | Intervention study | Self-help TB clubs to strengthen people affected by TB and patient-centred home visits to learn about the social network of the patient, identify strengths and weaknesses of the network, and plan activities to support the patient. The outcomes of the study were TB treatment outcome and internalised TB social stigma. |
