# Supplementary material for: A scoping review of patient-centred tuberculosis care interventions: Gaps and opportunities
Source: PLOS Glob Public Health. 2023 Feb 2;3(2):e0001357. doi: 10.1371/journal.pgph.0001357 (PMC10021744; doi:10.1371/journal.pgph.0001357)
Supplement: S4 Appendix — DR-TB = drug-resistant TB. (DOCX) [file pgph.0001357.s004.docx]

**S4 Appendix. Project reports and best practice documents included in the review.**

| **Organisation(s)** | **Title** | **Year** | **Description** |
| --- | --- | --- | --- |
| University Research Co. (URC) | Providing Comprehensive, Patient-Centred Care: A Conceptual Framework for Social Support of TB Patients | Sept, 2014 | The document provides a framework to better plan, manage, and coordinate delivery of social support and social protection services for people affected by TB, focusing on URC projects. It includes lessons from existing TB social support programs and offers guidance on how to develop a minimum package of effective social support and social protection services for TB patients. |
| USAID  TB CARE I  TB CARE II | Lessons learned from Best Practices in Psycho-Socio-Economic Support for Tuberculosis Patients | Nov, 2014 | The project draws lessons from best practices on sustainable systems for social support in National TB Programmes, with a focus on psycho-emotional and socio-economic support. |
| Médecins sans Frontières | Patient Support Interventions to Improve Adherence to Drug Resistant Tuberculosis Treatment: A Counselling Toolkit | 2015 | This counselling toolkit has been implemented with success in a community-based, decentralised patient-centred model of care for DR-TB in Khayelitsha, South Africa. The toolkit aims to standardise DR-TB counselling messages, ensure counselling quality, and promote improved outcomes for patients diagnosed with DR-TB. The toolkit guides dedicated DR-TB counsellors to offer individual and group counselling and psychosocial support for DR-TB patients and their families in structured counselling sessions that cover topics along the treatment journey, including treatment initiation, transition from intensive phase to continuation phase, treatment interruption, extensive drug resistance diagnosis, and DR-TB treatment failure. |
| USAID | Delivering Comprehensive  Supportive Care to People with Drug-resistant Tuberculosis: A Practical Toolkit | Sept, 2018 | The toolkit provides a standardized framework for supportive services to people affected by TB derived from international guidance and best practices from the field. It consists of an Introduction and Operational Toolkit which can be used to standardize and systematize the provision of supportive care services. By doing so, programmes can improve their ability to monitor and measure the outcomes of these services; gather evidence to evaluate their cost-effectiveness; and better integrate this work within their overall strategic planning and grant application processes. |
| USAID | Delivering Comprehensive Supportive Care Services to People with Drug-Resistant Tuberculosis: Report on Outcomes, Feasibility, Acceptability, and Cost-Benefit based on Pilot Implementation in China, Pakistan, South Africa and Ukraine | July, 2019 | This report describes the results of the pilot implementation of a supportive care package (outlined in the document above), in China, Pakistan, South Africa, and Ukraine. The care package framework is designed to address the challenges that people with DR-TB face in trying to complete treatment and be cured comprehensively, based on the literature, global guidance documents, and input from DR-TB patients. The framework is organized into four thematic areas and 13 elements. National TB Programmes can choose how they will address each element of care, according to the specific needs of the people they serve and what is feasible in the context of their health systems. |
| USAID  The Tuberculosis Coalition for Technical Assistance (TB\|CTA) | The TB Control Assistance Program (TB CAP) Patient Centred Approach | 2010 | The TB CAP developed a Patient Centred Approach package to facilitate consideration of patient needs in the planning and delivery of TB services. The package includes five tools that can be used individually or in combination, to address specific country needs: Patients’ Charter for Tuberculosis Care, QUOTE TB Light, Tool to Estimate Patients’ Costs, TB/HIV Literacy Toolkit, and Practical Guide to Improve Quality TB Patient Care. |
| USAID  TB CARE I | TB CARE I: Patient Centred Approach | n/a | The document outlines TB CARE I’s piloting of the TB CAP tools (referred to above) in collaboration with National TB Programmes in five countries: Cambodia, Indonesia, Mozambique, Nigeria and Zambia. Each country selected two or three tools for pilot implementation. The document details country experiences of implementing the tools and supports the argument that applying a patient-centred approach is an investment in changing health care culture. |
