# Supplementary material for: A scoping review of patient-centred tuberculosis care interventions: Gaps and opportunities
Source: PLOS Glob Public Health. 2023 Feb 2;3(2):e0001357. doi: 10.1371/journal.pgph.0001357 (PMC10021744; doi:10.1371/journal.pgph.0001357)
Supplement: S5 Appendix — DOT = Directly Observed Therapy. DR-TB = drug-resistant tuberculosis. LTFU = lost to follow-up. ART = antiretroviral therapy. (DOCX) [file pgph.0001357.s005.docx]

**S5 Appendix. Conference abstracts included in the review, grouped by implementation of patient-centred TB care.**

|  | **First author, year** | **Setting/**  **Country** | **Design** | **Implementation of patient-centred TB care** |
| --- | --- | --- | --- | --- |
| **Emotional and psychosocial support** | Balakrishnan et al. 2016 | India | Routine programme monitoring | Establishment of treatment support groups comprising socially responsible citizens and volunteers to provide social support to needy TB patients, ensuring access to information, free and quality services, and social welfare programmes, and supporting them to make decisions in their treatment journey and achieve treatment outcomes. |
|  | Baral et al. 2016 | Nepal | Qualitative methods | A psychosocial support intervention for DR-TB care including psychosocial counselling that considered the individual needs of people with TB, especially in terms of the level of social support required, as well as the experience of depression and anxiety, and included a health education component. |
|  | Li et al. 2015 | China | Intervention study | A patient-centred counselling strategy to prepare DR-TB inpatients for outpatient treatment and adherence upon discharge. |
|  | Li 2018 | China | Project evaluation | A supportive care package for DR-TB patients, including individualised care plans developed with and for patients, face-to-face and online education and counselling for patients and their families, a robust case management platform, and community-based care. |
|  | Islam et al. 2018 | Bangladesh | Pilot intervention study | Psychosocial support interventions for DR-TB patients consisting of bi-weekly support groups, expressive art therapy, edutainment and symbolic celebrations, and workshops on income generation activities. |
| **Decentralised TB care** | Casas et al. 2012 | Uganda | Community-based model of care | A decentralised community-based model of care for DR-TB treatment, including DOT, infection control, counselling, and psychosocial support. |
|  | Gelmanova et al. 2012 | Russia | Comparative study | A community-based model of integrated TB treatment which allowed patients the decision of place and time for DOT, close monitoring of side effects and comorbid conditions, access to specialist consultations including addiction experts and psychologists, and daily food sets and social support. |
|  | Geliukh et al. 2016 | Ukraine | Project evaluation using routine data | A project to support DR-TB patients at the outpatient stage of treatment, involving daily home visits by nurses to provide DOT, psychosocial support, manage side effects, along with twice monthly food packages. |
|  | Grant 2014 | South Africa | Pilot intervention study | An integrated programme in which facility-based counsellors provide treatment literacy on TB and HIV to patients, and community care workers provide weekly home-based support to TB/HIV patients and monitor their medication. |
|  | Phyo et al. 2019 | Myanmar | Mixed-methods study | A decentralised DR-TB treatment model implemented by community-based volunteers. |
| **Enhanced support for patients at risk of LTFU** | Fedotkina et al. 2011 | Russia | Project evaluation | A patient-centred TB care project that embraces clinical monitoring, psychological and social support, continuity of care, measures to reduce the harmful effects of alcohol abuse, community-based home care, the ‘Sputnik’ programme, individualised case management, and health education. |
|  | Mitra 2012 | India | Intervention study | Socioeconomic support for TB patients at risk of LTFU. Patients were linked to social welfare schemes, which included monthly provision of food grains, cash assistance for nutritional support, and snacks with DOT. |
|  | Mishustin et al. 2012 | Russia | Programme  evaluation | A patient-centred approach to accommodate the needs of DR-TB patients, including provision of daily food packages, treatment at home, inclusion of Red Cross volunteers, an anti-alcohol campaign, and the ‘Sputnik”’programme which provides intense treatment support and accompaniment to patients. |
|  | Taran et al. 2013 | Russia | Project model | A “Patient-Centred Accompaniment” project for high-risk TB patients, consisting of intensive psychological support, DOT at a location of the patient’s choosing, daily food sets, complementary side effects management, addiction care, and collaborative provider-patient relationships. |
| **Socio-economic relief** | Baral et al. 2010 | Nepal | Qualitative methods | Socioeconomic support to DR-TB patients, made critical due to the need for relocation to treatment centres given the Nepali terrain. |
|  | Fatima & Qadeer 2012 | Pakistan | Baseline assessment | An assessment of the preferences of DR-TB patients for social support, which included treatment supporters, food baskets, and transportation incentives. |
|  | Wingfield et al. 2014 | Peru | Pilot intervention study | A socioeconomic intervention to prevent TB that included household visits and community meetings (educational workshops and TB clubs) as well as cash transfers. |
| **Interventions aimed at health workers** | Missaut et al. 2014 | Cambodia, Indonesia, Mozambique, Nigeria, and Zambia | Project evaluation | Measured the changes attributed to implementation of the TB Control Assistance Patient Centred Approach Strategy and found that people with TB were more aware of their rights and responsibilities and were empowered to ask questions. Health workers were more attuned to patient perspectives on the quality of services, and of the cost barriers they face when accessing care. |
|  | Williams 2011 | n/a | n/a | A patient-centred training approach for health worker, developed by the Council of Nurses, and which has shown improved attitudes to patients, lower default rates, better case finding, and safer work environments. |
