# Supplementary material for: A scoping review of patient-centred tuberculosis care interventions: Gaps and opportunities
Source: PLOS Glob Public Health. 2023 Feb 2;3(2):e0001357. doi: 10.1371/journal.pgph.0001357 (PMC10021744; doi:10.1371/journal.pgph.0001357)
Supplement: Attachment — Submitted filename: Response to reviewers.docx [file pgph.0001357.s006.docx]

**Note.**

The line numbers referred to below correspond to the line numbers of the revised manuscript with track changes visible.

**Thank you.** We would like to thank the three reviewers for taking from their time to review our manuscript, for their constructive comments, questions, and suggestions, and their kind words and interest. We have carefully considered your comments and believe that the manuscript has been significantly strengthened as a result.

| **Reviewer #1** | |
| --- | --- |
| This paper aimed to conduct a scoping review of patient-centered TB interventions, which would be an important advance in the literature, given the importance of and increased focus (e.g., in global guidelines) on patient-centered TB care. Many of my comments/questions related to tensions between the inclusion and exclusion criteria listed in Table 1 | Thanks for recognising the importance of the topic. We appreciate your careful considerations and comments. We are aware of the tensions between the inclusion and exclusion criteria in some instances and have made revisions throughout the manuscript to acknowledge these tensions and define our criteria more clearly. |
| Table 1/lines 113-116: It is not clear what the rationale is for excluding “evaluations of interventions that aimed to improve adherence or treatment outcomes”. What if these interventions aimed to improve adherence or treatment outcomes by improving patient-centeredness? Although having interventions that respect individuals and focus on their needs is important in and off itself, isn’t improving outcomes an additional important potential benefit of more patient-centered care? It would be useful for the authors to clarify the rationale for this exclusion. | Our focus with the review was to find examples of how patient-centred care for TB has been implemented or put into practice. Our inclusion criteria focused on interventions aimed at improving the patient experience/patient-centred care.  Most often however, studies measured interventions to improve patient-centred care against a clinical outcome (e.g., adherence, reducing LTFU), or rather, aimed to improve adherence, LTFU, treatment outcomes by improving patient-centredness (giving patient-centred care an instrumental function, rather than pursuing it as an end in itself). For the purposes of the review, we included all such studies in our review (because it showed how patient-centred care for TB was put into practice), regardless of whether an intervention was found to have an impact on patient outcomes or not.  However, if an intervention aimed to improve adherence or treatment outcomes without a patient-centred mechanism, we would not include it in the review. Thus, the rationale for excluding ‘evaluations of interventions that aimed to improve adherence or treatment outcomes’.  We have edited section ‘2.1. Review inclusion and exclusion criteria’ and Table 1 to make this distinction more explicit for readers (see lines 104-129) and Table 1 on pages 6-7. |
| It would also be useful to better explain how it interacts/overlaps with the inclusion criterion “Evaluations of implemented patient-centred TB care enablers, such as psychosocial, material, or economic support”, as there were several instances throughout the paper in which it was not clear why certain articles were included or excluded and this likely relates to tension between the exclusion and inclusion criteria – such as: | Thanks for highlighting this. We have clarified our inclusion and exclusion criteria as detailed above (see section 2.1.). We also added the following line in section 2.1. (lines 126-128) to clarify a distinction we make between enablers and incentives: ‘We also excluded interventions that incentivised adherence with financial or other rewards as we considered incentives contrary to the principles and aims of patient-centred care.’  Certainly, we included articles if the authors said that they implemented psychosocial support or material/economic support to improve patients’ experience (with or without improving outcomes). If those same things were offered as an incentive to care initiation / adherence only, and not as a way to improve how patients experienced their care, we did not regard that as patient-centred, and rather as a focus on improving retention or LTFU. We agree that this was a difficult tension in our criteria, and often this was not obvious to us as an authorship team. We deliberated long on this distinction and had many discussions with our team of experts. What we present in the manuscript as the disaggregation of those two things is the outcome of these deliberations.  Having updated the inclusion/exclusion criteria to be explicit as above, we applied this to the studies we had found. We identified two research articles on socio-economic support that were previously excluded (see section 3.1.2., lines 232-248). We have included these articles and edited the manuscript, including the process diagram (Figure 1) to reflect this change. |
| The first few paragraphs of the results section include summaries of how interventions from several of the articles reduced LT FU – how was this not violating the exclusion criterion? | We believe this comment is addressed with our more explicit description of the inclusion/exclusion criteria in section 2.1., i.e., that we included interventions that aimed to improve outcomes (such as LTFU, adherence, treatment outcomes) if the mechanism to do so was through patient-centred care (see lines 109-112).  A key finding of our review is that few studies implement patient-centred care with the aim of improving the patient-centredness of the TB programme. Rather, PCC is implemented as a mechanism to improve outcomes (i.e., patient-centred care is used as an instrument for improving outcomes). |
| o It is not clear why relevant systematic review(s) are not discussed – such as the 2016 PLOS One article by van Hoorn et al (The Effects of Psycho-Emotional and Socio-Economic Support for Tuberculosis Patients on Treatment Adherence and Treatment Outcomes – A Systematic Review and Meta-Analysis). This review would seem to also evaluate “patient-centred TB care enablers, such as psychosocial, material, or economic support”? | We excluded systematic reviews in our scoping review because it was not in keeping with the aim of the study, which was to map, first-hand, the interventions that aim to implement patient-centred care for TB. The reasoning was that any single study/intervention included in a systematic review would be identified in our searches and would then undergo the screening process; including systematic reviews would lead to duplicate results.  We did however manually search the reference lists of systematic reviews identified in our searches, which is detailed in section 2.3. (lines 150-161). We also searched the reference lists of the full text articles included in the review. We added a line of clarification in this section to say that ‘These searches served as a proxy measure for the comprehensiveness of the review method’ (lines 160-161).  We found that this process of manually searching reference lists yielded only 1 additional research article not identified through our other database and Google searches. We have additionally included a line in the results section to make explicit how the 13 research articles were identified – clarifying that 12 were identified through the database searches and 1 through the manual searches of reference lists (see lines 179-180).  The systematic review by van Hoorn et al. which you refer to was included among the systematic reviews whose reference lists we hand-searched and the relevant studies were included. |
| Results section 3.2 on excluded articles: it is not clear why these articles were excluded. For example, several studies in this section are related to economic support – why does this not fit into exclusion criterion 3? Perhaps it could be helpful to explain why these articles were excluded in this section. | Thanks for highlighting this.  To clarify: for the purposes of the review we made the decision to include only interventions that explicitly focused on improving the patient experience (regardless of whether it was attached to improving patient outcomes such as LTFU, adherence, treatment outcomes for example) so that we could map how patient-centred care for TB had been implemented.  We also recognised that the line between the inclusion and exclusion criteria is somewhat blurred and there are some tensions, which is the reason for section 3.2. on ‘excluded research articles’. These were articles that did not fit our inclusion criteria (which was focused on finding examples of patient-centred care as per our criteria), in that they evaluated DOT, or, in the case of economic support, provided such support by way of incentive – which we consider contrary to patient-centred care principles. Even so, we consider it useful to have a section to outline some of the studies that we found but which did not make it into the review, as there are arguably lessons to be drawn from them. We have revised the paragraph to more clearly reflect this (lines 321-331).  We also have another section (section 3.5.) in which we discuss the findings with expert authors to get their thoughts and experiences on implementing patient-centred care for TB. These are valuable processes allowed for in scoping reviews, in that it offers reflection on the literature (using a certain set of keywords and criteria), what is excluded in that literature, and also to gain others’ insights on the findings of the review. Our discussion brings all of these findings together. |
| o Lines 270-271: “Three studies focused on mobile applications to support treatment adherence of people affected by TB (45–47).” Inclusion criteria #4 is “Mobile applications that aimed to facilitate and/or implement patient-centred care for TB, by, for example, providing information or psychosocial support” – so why were these articles excluded? | Similar to what we outline in the aforementioned point, we excluded these articles because the authors were using mHealth technologies without a patient-centred mechanism to improve the outcome (aimed at adherence). We included these studies in the ‘excluded articles’ section since there may be some lessons, but they do not offer an operationalisation of patient-centred care. We added details about these mHealth interventions to draw out some lessons more clearly and make the reason for their exclusion more explicit (lines 332-339). |
| In order for PRISMA scoping review guideline 8 to be met (“Present the full electronic search strategy for at least 1 database, including any limits used, such that it could be repeated”) I believe the exact search terms need to be provided (i.e., in an appendix). The current description on page 6 doesn’t provide enough information for the search to be replicated. For example, if I do a PubMed search with the terms “tuberculosis AND ("social" OR "socio-economic" OR "economic") AND support” limited to English-language articles between Jan 1 2005 and March 23 2020, 2532 articles are returned, which is far more than the 853 articles mentioned in Figure 1. | Thank you for highlighting this. We have added our full electronic search strategy for one of the databases as S2 Appendix and referred to this in text in lines 138-139. |
| Lines 280-283: “These interventions reinforce the importance of embedding patient centredness in TB services and offer useful tools for implementing patient-centred care principles. However, they do not introduce novel evaluations of interventions which have not already been described in the review of published articles.” The introduction and methods leave the impression that the purpose of the article is not just to evaluate patient-centered interventions (indeed, this seems to fit with the broader purposes of a scoping review and justifies the authors decision not to conduct a systematic review). But in this and some other places in the results, it seems as though only papers that reported some sort of impact/outcome are discussed. Were papers that did not include impact on adherence/treatment outcomes/some other outcome explicitly excluded? If so, this should be clarified in the methods. If not, it would still seem relevant to this review to discuss the interventions discussed in these project reports and best practice documents, even if the impact of these interventions was not evaluated. | Thanks for this comment and query. Our study was certainly not to review only papers that documented impact or outcome (as detailed in our responses to your queries on the inclusion/exclusion criteria above – see section 2.1.).  As mentioned in lines 102-129 – in our review we aimed to find interventions that implemented patient-centred care to improve the patient-centredness of a TB service or programme, considering it the gold standard. It is a key finding of this review that nearly all of the research on patient-centred TB care that we found, patient-centred care was used as a means to improve adherence and treatment outcomes, rather than an end in itself. In all but three examples, adherence and treatment outcomes were measured against a patient-centred intervention.  We believe that more detailed description of the included studies can help to better sketch the patient-centred TB care interventions that we found in our review, as their current descriptions include a focus on the outcomes measured. We have added such details throughout the results section (section 3, starting line 176). Some of these details had previously been omitted on the basis of word count.  In the absence of indicators, we have also revised Figure 4’s caption to reflect the limitation of patient-centred care indicators (lines 524-529) more clearly. |
| Section 3.4 – it would be useful to categorize the interventions found in the conference abstracts using the same categorizations that were used for the published studies (in Section 3.1) or the categorizations used in Figure 4. It would also be useful to add these categorizations to the appendix tables that summarize the articles. | Thanks for this good suggestion. We have organised the abstracts using the same categories as used for the published studies and have updated the manuscript text to reflect this (lines 359-369). |
| A point emphasized in the discussion is the need to track “patient-centred care outcomes” rather than just adherence/LTFU/treatment outcomes. Some examples of articles that do this are mentioned, but very few details are provided on what the outcomes were that were measured. Given the dearth of studies measuring these outcomes, most readers of this paper may not be very familiar with the concept of patient-centred care outcomes. The authors should consider discussing these outcomes in more detail (e.g. for example in the results/through a table or appendix table) – potentially including not just those that have been evaluated in studies but also those that were proposed (for example in the Arsenault, Order-DeWan, and Kruk article). Is there a reason why these outcomes are not included in Figure 4? | Thanks for raising this important point and highlighting how it could make patient-centred care indicators more concrete for readers. We found only three articles in our review that included measurements of the impact/effect of the intervention independent of treatment outcomes. We have included specific mention of these three articles and their indicators in the discussion section (lines 410-419).  We have also included more detailed discussion of the limitations of specific patient-centred care indicators in the discussion, drawing on the article by Arsenault et al. and the patient-centred approach package implemented by USAID’s TB Control Assistance programme (lines 436-472). |
|  |  |
| **Reviewer #2** | |
| The focus on "patient" or "people" centered care is an important area of analysis. The existing publication may be further strengthened with more in-depth consideration of human rights and gender related barriers to accessing quality TB services. Noting that a TB CRG Assessment has been completed in South Africa and that a global level analysis of human rights and gender related barriers has also been published in a peer reviewed context as well (<https://www.hhrjournal.org/2021/12/building-the-evidence-for-a-rights-based-people-centered-gender-transformative-tuberculosis-response-an-analysis-of-the-stop-tb-partnership-community-rights-and-gender-tuberculosis-assessment/> ) . | Thanks for this useful comment. We make brief mention of the gap in existing interventions in which we refer to a lack of distinction ‘between how men and women may be differently affected by TB or challenged to engage in TB treatment’. We have further referenced the CRG Assessment to bring attention to these dynamics in TB responses (lines 480-482). |
| Finally it may be useful to also look at the recently released Global Plan to End TB 2023-2030 and draw further on the practical elements of person centered care. | Thank you for pointing us to the latest document. We have referenced this document in our discussion on a few occasions and engage with it more directly in lines 482-487.  Unfortunately, and this is part of the reasons for the review, patient/person centred care is poorly operationalised in guidance and policy documents. This includes the Global Plan to End TB, which reinforces the importance of patient-centred care, but has limited guidance on how to actually implement it in NTPs. |
|  |  |
| **Reviewer #3** | |
| The submission of the manuscript is timely and critical in view of the operational lack of emphasis on what is a critical pillar in the end TB strategy. The period included of 2005 to 2020 is relevant particularly since it included the recognition of the compounded problems of an ever-increasing situation of DR TB. | Thank you for recognising the importance and timeliness of our study. |
| The methods and inclusion criteria are sound and the definitions and findings articulated well. | Thanks for this comment. |
| Perhaps the search needed to include policy documents of large TB control programmes like the NTEP to demonstrate which issues of patient centric care are highlighted there and triangulate these with implementation studies in that context. The contacting of authors for implementation insights is also commendable. | This review was conducted in South Africa and was commissioned by the South African National TB Think Tank. The aim of this work was to inform how to operationalise patient-centred TB care in this setting. One of the project deliverables was a policy brief outlining the findings of the review.  From our own experience, and what we gleaned from interviews, there is an emphasis in policy documents that TB programmes should be patient-centred, but there is little information on how to do it in practice.  As such, in this manuscript our focus is on mapping how patient-centred TB care has been operationalised, and to highlight gaps and opportunities. Your suggestion to triangulate policy recommendations with what has been done in practice is useful, but was beyond the scope of our methodology to pursue our aim. |
| One of the seminal studies on PCC from Peru was the Lima study of Paul Farmer on DR TB patients. This and the Papworth study in the UK could have been mentioned as a background or at least in the Discussion. | Thank you. We could not find published evaluations of either study using our search criteria (which include reference lists of reviews and included manuscripts). If the reviewer could kindly provide the citations, we will revert. |
| The manuscript may like to include which of the interventions in PCC have been actually incorporated in policy. | This review was conducted to move towards policy, and this is what we report on in the findings. Currently the details on how to make patient-centred TB care real are lacking – we agree that it would be useful to conduct a review that makes the link between policy and practice, but it was beyond the scope of the aims of this review. |
| Line 267 mentions about the role of financial support. Maybe good here to expand a little citing the reasons why it alone is not sufficient for offsetting catastrophic costs. | Thanks for this comment. We have included more detailed description of the study in question in lines 237-243. |
| The authors may also like to provide emphatic guidance to readers as to which approach for PCC yields the most optimal patient outcomes and the authors have repeatedly stressed the importance of measuring the outcomes. | We agree that this would be incredibly useful. In this *scoping* review, rather than a synthesis of evidence, what we first charted is how patient-centred TB care is operationalised in the literature. What we found is that patient-centred TB care is being operationalised towards improving treatment outcomes, rather pursued as an end in itself. We have included more detailed discussion of patient-centred TB care outcomes, highlighting the three studies included in our review which include measures other than patient outcomes (lines 410-419), and discussing a published framework and a patient-centred care approach strategy by USAID (lines 436-472). We have also adapted the caption of Figure 4 to better reflect the limitations of patient-centred care indicators/outcomes, lines 524-529.  Currently it is premature to know what works best in terms of improving patient-centred TB care, because hardly any interventions that aim to improve and measure the patient experience has been implemented to date. |
| Studies on delivery of integrated comprehensive care in adults and children is another important point mentioned by the authors which needs mention of the specific studies that were undertaken for arrival at a conclusion. | We make mention of the gap in patient-centred care interventions focused on children and adolescents (lines 476-478, 484). Only one of the interventions reviewed included consideration of the specific needs of these patient categories/groups. As such there are no specific studies to mention further (rather a lack of studies/interventions) that led us to this conclusion. We remark on this as a notable gap in the intervention literature for patient-centred care. |
| Similarly has a comparative analysis been done on the effect of virtual/remote or in person support. Studies on patient preferences maybe highlighted. | Such work has not been done by us, and it is not within the scope of the review. |
| The review should also question whether the concept of patient-oriented care (PCC) has been ingrained in the front line workers in the various countries as a part of their in service or pre service training. | Thanks for this suggestion. In the literature (and in our experience), patient-centred care is a poorly understood concept in health services overall, and in LMIC specifically. We mention in our discussion that for patients to be engaged as partners in care, work with health workers is needed (lines 492-494), and we further added in the discussion that ‘how frontline health workers internalise and support patient-centred care principles ultimately influences how guidelines and policies become practice’ (lines 494-496). |
